# Infant cardiac MRI using oscillatory ventilation: safe and effective

**DOI:** 10.1186/1532-429X-14-S1-P122

**Published:** 2012-02-01

**Authors:** Alicia H Chaves, George M Hoffman, Joseph Cava, Pippa Simpson, Margaret Samyn

**Affiliations:** 1 Pediatrics, University of Maryland, Baltimore, MD, USA; 2 Pediatrics, Medical College of Wisconsin, Milwaukee, WI, USA

## Summary

We hypothesize that use of the high frequency oscillatory ventilation (HFOV) during infant cardiac magnetic resonance imaging (CMRI) is safe, faster, and results in image quality equivalent to that obtained using conventional ventilation with breath-holding.

## Background

Cardiac magnetic resonance imaging (CMRI) for infants and young children typically requires sedation. General anaesthesia can eliminate motion artefact and permits controlled ventilation with breath-holding during imaging to limit respiratory artefact, but this technique typically requires frequent changes in respiratory support that may impair pulmonary function, and pauses in image acquisition during recovery from breath-holding. HFOV provides ventilation with near-constant mean airway pressure, limited tidal volume, and minimal movement of chest wall and diaphragm, thus obviating the need for breath-holding during imaging. We have recently described a hybrid HFOV-anaesthesia delivery system for use during CMRI [Hoffman, 2010], but image quality has not been addressed heretofore.

## Methods

Clinical data were collected for 8 infants, who underwent CMRI with HFOV and 8 age and size matched controls, who underwent CMRI with conventional ventilator (CONV) and breath hold technique. Data included demographic information, cardiac diagnoses, vital signs at the start and end of the exam, adverse events, and scan acquisition time. Cine steady state free precession (SSFP) or gradient ECHO imaging (GRE) and T2 turbo spin echo (TSE) images were reviewed for image quality, using a scale of 1 (poor quality) to 4 (excellent quality) by two cardiologists blinded to type of ventilation. Significance cut-off for reported difference was p<0.05.

## Results

Complex congenital heart diseases were similarly represented in the two groups (Table 1). There were no significant differences in age, weight, body surface area, or ASA score between the two groups. There was no significant difference in average image quality for cine short axis or black blood imaging (Table 2). The total CMRI scan time was not significantly different between groups, but the short axis cine stack was acquired more quickly in the HFOV group (1.8± 0.8 vs. 5.0 ± 3.6 minutes). The mean airway pressure was higher (11.9 ± 1.0 vs 8.9 ± 1.5 mmHg) and fiO2 lower (0.28 ± 0.05 vs .57 ± 0.21) with HFOV. There were no adverse events (AE) in the HFOV group, but scans were terminated early for 2 patients in the conventional ventilator group due to oxygen desaturation, with one requiring post-procedure mechanical ventilation. Other vital signs (temperature, heart rate, and blood pressure) were similar before and after CMRI, but 4/8 patients managed with CONV required increased fiO2 after scanning compared with 0/8 managed with HFOV.

**Table 1:** Demographics

	HFOV (N = 8 )	CONV (N = 8 )
Gender (females)	6	6
Age (weeks) median (range)	6.0 (0.1 - 29.9)	8.0 (0.3 - 33.7)
Weight (kg) median (range)	3.84 (2.5 - 6)	4.25 (2.9 - 7)
BSA (meters squared) median (range)	0.22 (0.17 - 0.32)	0.24 (0.18 - 0.33)
Diagnosis (number of patients) Single ventricle, unoperated Single ventricle, palliated Two ventricles , unoperated Two ventricles, palliated	3 1 2 2	1 2 5 0
ASA score (mean) (median, range)	3.38 (4.0, 2.0 - 4.0)	3.13 (3.0, 2.0 - 4.0)

**Table 2:** CMRI data

	HFOV (N = 8)	Conventional (N = 8)
MRI scan time (minutes), mean (median, range)	69.5 (65.0, 42.0 - 101.0)	78.1 (76.0, 39 - 103.0)
Short axis time (minutes), mean (median, range)	1.8* (1.5, 1.0 - 3.25)	5.0 (3.6, 2.4 - 13.4)
Cine quality score, mean (median, range)	2.6 (2.5, 2.0 - 3.5)	2.5 (2.5, 2.0 - 3.0)
TSE quality score, n = 7 mean (median, range	2.9 (3.0, 2.0 - 4.0)	3.0 (3.0, 2.0 - 3.5)
Adverse events	0%	25%

* p <0.01.

## Conclusions

HFOV during CMRI is feasible and well tolerated. Image quality is equivalent to that obtained with conventional ventilation with breath-holding technique and allows shorter cine scan times for some sequences. Although not statistically significant, the HFOV group tended to have lower weight, higher ASA, and more unoperated single ventricle disease; thus, HFOV technique may be especially suitable to smaller, sicker patients.

## Funding

None.

